# Analysis of (co) variance components and estimation of breeding value of growth and production traits in *Dahlem Red* chicken using pedigree relationship in an animal model

**DOI:** 10.1371/journal.pone.0247779

**Published:** 2021-03-03

**Authors:** U. Rajkumar, L. Leslie Leo Prince, K. S. Rajaravindra, S. Haunshi, M. Niranjan, R. N. Chatterjee

**Affiliations:** ICAR-Directorate of Poultry Research, Rajendranagar, Hyderabad, Telangana, India; University of Nicolaus Copernicus in Torun, POLAND

## Abstract

Variance and covariance components of growth and production traits were analyzed employing REML animal model to assess the *Dahlem Red* (PD-3) chicken population for direct additive genetic, maternal effects and to estimate the estimated breeding value (EBV), genetic parameters, genetic trends and rate of inbreeding (ΔF) utilizing seven generation’s data. The generation and hatch had significant (P≤0.01) effect on the body weight at 0 day (BW0), 2 (BW2), 4 (BW4) and 6 weeks (BW6) and shank length at six weeks of age (SL6). The average least squares means (LSM) for BW6 and SL6 were 273.93±0.62 g and 53.97±0.05 mm, respectively. All the production traits were significantly (P≤0.01) influenced by generation and hatch. The average LSM for age at sexual maturity (ASM), egg production up to 40 weeks (EP40) and egg mass up to 40 weeks (EM40) were 168.82±0.25 d, 72.60±0.41 eggs and 4.21±0.07 kg, respectively. Model 5 with additive direct, maternal genetic, maternal permanent environmental and residual variance components was the best for BW0, BW2 and BW4 based on the AIC values obtained in WOMBAT. Model 4 was the best model for BW6, SL6, ASM, EP40 and EM40 with additive direct, maternal permanent environmental and residual variance components. Maternal effects were higher during early age, decreased with age, and remained present until 20 weeks of age. The heritability (h^2^) estimates were low to moderate in magnitude for all the growth traits and ranged from 0.02±0.03 to 0.19±0.03. The maternal heritability was high at hatch (0.35±0.06), decreased gradually until 4^th^ week (0.02±0.01) and ceased afterwards. The heritabilities of EP40 (0.11±0.03) and EM40 (0.12±0.04) were low. The direct additive genetic correlations (r_a_) between BW2, BW4, BW6 and SL6 were high and positive (P≤ 0.05). The additive genetic and maternal permanent environmental correlation between EP40 and EM40 were high and positive (P≤ 0.05). The EBV of EM40 was significant (P≤ 0.05) with 0.48 kg/generation in PD-3 chicken at the end of the seventh generation. The EBV of EP40 showed an increasing trend with a genetic gain of 1.87 eggs per generation. The average inbreeding coefficient of the population was 0.019 and average ΔF was 0.007 over the last seven generations of selection. The EBV trends for primary and associated traits showed linear trends in the desired direction and negligible inbreeding.

## Introduction

Poultry production has grown exponentially in structure and operation from a traditional backyard activity into a major commercial agri based industry over the years. Development of high yielding layer (310–340 eggs) and broiler (2.4–2.6 kg at 6 weeks) varieties together with standardized practices on nutrition, housing, management and disease control [1] have contributed to spectacular growth rates in the egg (8.5% per annum) and broiler production (7.8% per annum) in India [2]. Backyard poultry contributes about 17% (17.09 billion) to the total egg production (103.32 billion) of the country and chicken meat is 50% (4.06 MMT) of the total meat production (8.10 MMT) in India [2]. India ranks 3^rd^ in egg production and 5^th^ in meat production in the world, but the availability of chicken products is far below the recommended levels. The per capita availability is about 79 eggs and 3.2 kg chicken meat per annum against the recommended level of 180 eggs and 10.25 kgs meat as per the Indian Council of Medical Research. Backyard poultry farming with improved chicken varieties such as *Vanaraja*, *Gramapriya*, *Srinidhi*, and *Rajasree* is promoted by the Government as it is one of the proven technologies for poverty alleviation and nutritional security in rural and tribal areas of the country. A wide gap exists between the availability and requirement of chicken products, thus providing an opportunity to expand the poultry farming in the commercial as well as the backyard sectors. Since commercial poultry has inherent difficulties for expansion in rural and tribal areas, the backyard poultry has bright scope in these areas. Promoting the backyard poultry farming among the rural masses is going to increase its share in total poultry production apart from providing healthy animal protein in terms of eggs and meat. *Dahlem Red* (PD-3) is the female parent of *Gramapriya*, an improved backyard chicken layer variety propagated across the country. The improvement in egg production, egg mass, growth and other economic traits in the terminal cross is being achieved through the application of selection in the parent lines.

The estimated breeding value (EBV) determines the rate of improvement in the primary trait of selection. Variability in the population is the basis for any genetic improvement program in livestock and poultry. Genetic improvement is determined by the response to selection in the primary as well as other correlated traits of economic importance. Thorough knowledge on the genetic basis, gene action and the environmental effect is essential for planning breeding programs for genetic improvement of the desired traits in poultry. Selection response of trait depends on the population size, gene frequency, mutation, allelic fixation, random drift and physiological limits [3]. The magnitude and direction of correlated responses play a major role in developing effective breeding strategies for improved productivity, more so among positively correlated traits due to linkage and pleiotropy [4].

Partition of variance into every possible source and their interaction effects reduces the error variance and minimizes over-weightage to some components, leading to precision in the estimates. Animal model accommodates additional components of variance, which makes the estimates less biased by confounding environmental variation and precise modeling [5]. The traditional models ignore the maternal and permanent environmental effects in chicken leading to overestimation of additive genetic variance resulting in high h^2^ estimates. The maternal effect is defined as the situation where the phenotype of the offspring is determined not only by the environment and its genotype, but also by the genotype and environment of their mother. A maternal effect is a situation wherein traits are influenced by the genotype and environment of the mother. Maternal effects are important in the development and expression of the economic traits due to genetic or environmental differences between dams or by the combination of genetic or environmental differences [6, 7]. The egg weight is a major factor contributing to maternal effects for body weight, however, others like hatch weight, incubation conditions, nutrition, etc. may also play a role. Correlations of egg weight with hatching weight and with the subsequent body weight of chicks may also reflect maternal effects. The inclusion of maternal effects in the model reduces the bias and increases the precision of genetic parameters [8]. Many authors estimated the genetic parameters without considering the maternal effects in chicken [9–16]. Some publications with additive, maternal and permanent effects on various economic traits using animal model [4, 17] and diallel analysis [10, 18] were reported in chicken. However, studies with large data sets over the generations utilizing an animal model in PD-3 line are limited from India. The analysis of data using traditional models overestimated the genetic parameters and lead to inadequate inferences affecting the long term breeding experiment especially in India.

Therefore, the present study was taken up with an aim to estimate the breeding value, genetic trends of economic traits, genetic (additive and maternal) and non-genetic environmental effects and performance of important economic traits in (PD-3 line using Average Information Restricted Maximum Likelihood (AIREML) animal model.

## Materials and methods

The study was carried out at the experimental poultry farm of ICAR-Directorate of Poultry Research, Hyderabad, Telangana, India. Hyderabad is in Deccan plateau placed between 17°23’ N and 78° 28’ E at 500 m from mean sea level. The climate is hot tropical with maximum temperature ranging from 20°C in winter to 45°C in summer.

### Ethic and biosafety statement

The experiment was approved by the Institutional Animal Ethics Committee (IAEC/DPR/17/4). The manuscript does not contain clinical studies and patient data.

### Population and management

PD-3 population was selected over the generations [19, 20] for higher egg production and egg mass. It is the female parent line of *Gramapriya*, a popular brown egg layer suitable for rural poultry farming developed by ICAR-Directorate of Poultry Research. PD-3 line was developed from *Dahlem Red* breed. The work on the development of PD-3 line as a female line for production of egg type rural poultry was started in 1998–99. A pedigreed population of this line was produced for the first time (S-0) in 2000–01. After recording the performance for 6 generations, a random mating program using pooled semen for two generations was followed. The variability in the existing population was increased subsequently with the addition of *Dahlem Red* population from Anand Agriculture University, Anand, Gujarat, India. The base population was stabilized for two generations with random *inter se* mating and the third generation was considered as the base population with the initiation of pedigreed mating and selection. The population was selected for higher 40 week egg mass (EM40) during the last seven generations.

About 3500 to 4000 healthy chicks were produced in 7–8 hatches in each generation in a pedigreed mating with 50 sires and 250 dams. These chicks were wing banded and reared on deep litter in an open sided poultry house under standard management. About 600 females and 200 males were housed in individual cages in each generation at 12–14 weeks of age. The chicks were fed *ad-libitum* layer starter diet (2800 Kcal: ME and 18%: CP) based on maize-soybean meal up to 6 weeks of age in each generation, with layer grower ration (2700 Kcal: ME and 18% CP) until 16 weeks of age and with layer breeder ration (2650 Kcal: ME, 16.50% CP and Calcium: 3.5%) until 40 weeks of age. The chicks were vaccinated against Marek’s disease (MD) on the 1^st^ day; Newcastle disease (ND) on the 5^th^ and 14^th^ days; infectious bursal disease (IBD) on the 24^th^ day and fowl pox (FP) on the 42^nd^ day, ND with R_2_B at the 9^th^ week, infectious coryza at the 12^th^ week and ND, IBD and infectious bronchitis (IB) combined vaccine at the 18^th^ and the 40^th^ weeks of age.

### Data and traits

Data on growth and production traits of 16,292 PD-3 birds descended from 336 sires and 1076 dams collected over seven generations (S1 to S7) from 2012–13 to 2018–19 were utilized in the study. In each generation, juvenile body weight at 0 day (BW0), 2^nd^ (BW2), 4^th^ (BW4) and 6^th^ (BW6) week of age; shank length at 6^th^ week (SL6); adult body weight at 20^th^ (BW20) and 40^th^ (BW40) weeks were measured. The body weight was measured to 0.1g accuracy using digital balance, while shank length was measured to the nearest of 0.01mm accuracy using digital Vernier Calipers. Age at sexual maturity (ASM); egg weight at 28 (EW28) and 40 (EW40) weeks; egg production (EP40) and egg mass (EM40) up to 40 weeks of age were recorded. The weight of eggs was recorded using a digital balance to an accuracy of 0.01g. The data were not recorded on BW0 in S-3 and S-7; BW2 in S-6 and hatch 8; BW40 in S-7 generation. The descriptive statistics of growth and production data are presented in Table 1.

**Table 1:** Characteristics of data on growth and production traits in the PD-3 line.

Trait^1^	No. of birds with records	No. of sires	No. of sires with records and progeny	No. of dams	No. of dams with records and progeny	Mean	SD
**BW0, g**	10606	276	144	744	468	36.40	3.72
**BW2, g**	12917	315	153	919	505	79.02	19.63
**BW4, g**	14271	331	231	1025	890	158.86	43.87
**BW6, g**	14433	335	286	1029	934	280.35	73.52
**SL6, mm**	14199	335	283	1028	917	54.31	6.46
**ASM, d**	4261	328	-	887	807	167.68	16.69
**BW20, g**	4059	327	-	888	753	1323.28	196.87
**BW40, g**	3216	277	-	683	590	1691.04	231.55
**EP40, no**	4277	328	-	893	817	74.07	26.26
**EM40, kg**	3287	324	-	835	698	4.289	1.267
**EW28, g**	3467	325	-	863	710	49.68	4.20
**EW40, g**	3290	324	-	835	699	53.84	4.62

^**1**^BW0: Day old body weight, BW2: 2^nd^ week body weight, BW4: 4^th^ week body weight, BW6: 6^th^ week body weight, SL6: 6^th^ week shank length, BW20: 20^th^ week body weight, BW40: 40^th^ week body weight, ASM: Age at sexual maturity, EP40: 40 weeks egg production, EM40: Egg mass up to 40 week, EW28: 28^th^ week egg weight, EW40: 40^th^ week egg weight.

### Statistical analysis

Variance and covariance components were estimated by average information restricted maximum likelihood (AIREML) fitting an animal model [21]. Data were first analyzed by least squares analysis of variance (SPSS 12) to identify the fixed effects to be included in the model [22]. Two statistical models were used for identifying the significant effects on the traits. For BW0, BW2, BW4, BW6, SL6, ASM, BW20, BW40, EP40 and EM40, the statistical model included the fixed effect of generations (7 levels) and hatch number (8 levels). Only significant effects (P≤0.05) were included in the models, which were subsequently used for the genetic analysis. Convergence was assumed when change of value of the natural logarithm of the likelihood function in two consecutive iterations was lower than 5× 10^−4^. Univariate animal models were fitted to estimate (co)variance components for all the traits. The six single–trait models used to estimate variance components were defined as follows:

$$y=X\beta +Z_{a}a+\varepsilon$$ (1)

$$y=X\beta +Z_{a}a+Z_{m}m+\varepsilon \;with\;Cov\;(a_{m},\;m_{o})=0$$ (2)

$$y=X\beta +Z_{a}a+Z_{m}m+\varepsilon \;with\;Cov\;(a_{m},\;m_{o})=A\sigma_{am}$$ (3)

$$y=X\beta +Z_{a}a+Z_{pe}pe+\varepsilon$$ (4)

$$y=X\beta +Z_{a}a+Z_{m}m+Z_{pe}pe+\varepsilon \;with\;Cov\;(a_{m},\;m_{o})=0$$ (5)

$$y=X\beta +Z_{a}a+Z_{m}m+Z_{pe}pe+\varepsilon \;with\;Cov\;(a_{m},\;m_{o})=A\sigma_{am}$$ (6)

Where **y** is the vector of records; ***β*, a, m, pe** and ***ε*** are vectors of fixed, direct additive genetic, maternal additive genetic, permanent environmental effects of the dam, and residual effects, respectively; with association matrices X**, Z**_**a,**_ **Z**_**m**_ and **Zpe**_;_ **A** is the numerator relationship matrix between animals; and σ_am_ is the covariance between additive direct and maternal genetic effects. Assumptions for variance (**V**) and covariance (**Cov**) matrices involving random effects were **V**(a) = **A**σ^2^_a_, **V**(m) = **A**σ^2^_m,_ **V**(c) = **I**σ^2^_c_, **V**(e) = **I**σ^2^_e_, and **Cov**(a,m) = **A**σ_am,_ where **I** is an identity matrix and σ^2^_a,_ σ^2^_m,_ σ^2^_c_ and σ^2^_e_ are additive direct (a), additive maternal (m), maternal permanent environmental (c) and residual variances (e), respectively. The heritability (h^2^) was calculated as h^2^ = σ^2^_a_/σ^2^_p,_ where σ^2^_p_ = σ^2^_a_ + σ^2^_m_ + σ_am_ + σ^2^_c_+ σ^2^_e_. The m^2^, r_am_ and c^2^ were calculated as m^2^ = σ^2^_m/_ σ^2^_p_, r_am_ = σ_am_/ σ^2^_p_ c^2^ = σ^2^_c/_ σ^2^_p,_ respectively. The total heritability (h^2^_t_), was calculated using the formula h^2^_t_ = h^2^ + 0.5m^2^ + 1.5mr_am_h [23]. Akaike’s Information Criterion (AIC) was used for selecting the best model among the tested models [24]. The model with lowest AIC value was chosen as most appropriate model and used to study the genetic parameters.

The best models from the single trait analyses were combined with appropriate (co)variance between random effects in the model for the bivariate analysis. The best model identified for a specific trait using likelihood ratio test was only used for the bivariate analysis. The estimates of genetic parameters i.e., genetic, phenotypic and environmental correlations between different economic traits were obtained by AIREML fitting an animal model. To test the significance of the genetic covariance, the full model was compared with the model in which genetic covariance was zero (COV_A_ = 0). Significance of maternal permanent environmental covariance was also tested accordingly (COV_C_ = 0). [25]. Significance of phenotypic correlations was tested by the hypothesis test to decide whether the value of the correlation coefficient was significantly different from zero [26].

The EBV obtained from the best single trait model suited for each trait was used to plot the genetic trend. The genetic trend was estimated by regression of the EBV of the females that contributed in each generation for the trait under selection (EM40) and the other production traits under study [26]. The EBV of contributed individuals was utilized for estimating the genetic trends of growth traits.

## Results

### Growth and production traits

The least squares means (LSM) for body weight up to six weeks of age and SL6 are presented in Table 2. Generation and hatch had significant (P≤0.01) effect on the body weight (BW0, BW2, BW4 and BW6) and shank length (SL6) in PD-3 line. The BW6 and SL6, the important juvenile traits showed an increasing trend over the generations. Hatch 1 recorded significantly (P≤0.01) higher body weights and shank length. The interaction between the generation and hatch was significant (P≤0.01). The LSM for interaction effects of growth traits is presented in Table 3. The overall average LSM for BW6 and SL6 were 273.93±0.62 g and 53.97±0.05 mm, respectively.

**Table 2:** Least squares means of juvenile growth traits of PD-3 line.

Particulars	Body weight, g				Shank length, mm
	0 day	2 wks	4 wks	6 wks	6 wks
**Overall LSM**	36.41±0.04 (10606)^1^	77.45±0.17 (12917)	153.56±0.36 (14271)	273.93±0.62 (14433)	53.97±0.05 (14199)
**Generation**	**	**	**	**	**
**1**	37.28±0.12^e^ (1050)	64.74±0.73^a^ (537)	123.66±1.58^a^ (585)	226.48±2.25^a^ (880)	52.68±0.19^b^ (883)
**2**	35.41±0.08^a^ (2284)	73.36±0.34^b^ (2202)	142.73±0.80^b^ (2167)	247.51±1.42^b^ (2017)	51.33±1.12^a^ (2043)
**3**	**-**	91.39±0.32^d^ (2681)	178.88±0.76^d^ (2577)	282.27±1.35^e^ (2346)	53.68±0.12^b^ (2147)
**4**	36.79±0.07^c^ (2748)	74.36±0.32^b^ (2441)	141.89±0.93^b^ (1738)	265.64±1.45^c^ (2080)	51.30±0.12^a^ (2043)
**5**	37.11±0.09^d^ (2188)	79.49±0.37^c^ (2182)	169.17±0.88^c^ (2165)	320.52±1.49^g^ (2184)	57.24±0.13^c^ (2188)
**6**	35.61±0.08^b^ (2336)	**-**	141.21±0.86^b^ (2198)	269.66±1.49^d^ (2165)	53.70±0.13^b^ (2149)
**7**	**-**	79.52±0.30^c^ (2874)	174.18±0.72^c^ (2841)	312.25±1.25^f^ (2761)	57.22±0.11^c^ (2746)
**Hatch**	**	**	**	**	**
**1**	36.78±0.09^e^ (1727)	85.69±0.46^e^ (2247)	170.46±0.81^f^ (2314)	295.39±1.29^e^ (2609)	57.60±0.11^f^ (2622)
**2**	36.71±0.09^de^ (1901)	86.75±0.38^e^ (1884)	162.18±0.81^e^ (2321)	270.87±1.56^c^ (2157)	53.46±0.13^cd^ (2161)
**3**	35.98±0.09^cd^ (2118)	73.65±0.34^c^ (2206)	156.12±0.81^e^ (2616)	284.87±1.41^e^ (2507)	54.41±0.13^e^ (2171)
**4**	36.50±0.10^e^ (1879)	77.91±0.36^d^ (2657)	148.12±0.84^d^ (2760)	271.44±1.45^d^ (2683)	53.52±0.12^cd^ (2692)
**5**	36.97±0.08^f^ (2202)	71.01±0.34^b^ (2649)	153.22±0.82d^e^ (2477)	259.89±1.30^b^ (2766)	52.56±0.11^b^ (2855)
**6**	35.69±0.23^bc^ (258)	64.62±0.84^a^ (530)	131.05±1.74^b^ (646)	270.46±3.04^d^ (621)	53.43±0.25^de^ (626)
**7**	35.78±0.21^b^ (367)	69.71±0.91^b^ (744)	145.82±1.65c (990)	255.37±2.89^b^ (945)	52.79±0.24^c^ (927)
**8**	34.76±0.29^a^ (156)	**-**	113.35±3.03^a^ (147)	249.75±5.23^a^ (145)	51.39±0.44^a^ (145)
**Gen x Hatch**	**	**	**	**	**

^**1**^Values in the parentheses are number of observations.

Means with same superscripts do not differ significantly

* (P<0.05)

** (P<0.01)

**Table 3:** Least squares means of interaction effects between generation and hatch of juvenile growth traits.

Gen.	Hatch	Body weight, g				Shank length, mm
		0 day	2 wks	4 wks	6 wks	6 wks
1	1	38.40±0.23^lm^	75.58±2.16^ij^	-	215.59±4.31^b^	65.63±0.37^x^
	2	38.70±0.29^m^	-	-	238.24±6.36^ef^	49.26±0.55^b^
	3	36.82±0.31^g-j^^1^	-	143.13±3.32^g-j^	231.37±5.82^d-e^	51.59±0.49^e-g^
	4	37.06±0.33^h-j^	57.96±1.48^a^	107.57±3.68^a^	199.41±6.33^a^	49.08±0.54^b^
	5	36.48±0.26^g-i^	70.24±1.15^f^	123.75±2.74^cd^	218.90±4.75^bc^	50.81±0.40^de^
	6	36.66±0.33^g-i^	58.91±1.15^ab^	118.02±3.63^bc^	221.66±6.43^b-d^	49.44±0.53^bc^
	7	36.83±0.37g-j	61.00±1.71^bc^	125.82±4.14^de^	260.17±7.09^g-j^	52.96±0.60^h-j^
2	1	34.97±0.18^a-c^	83.72±0.77^mn^	183.64±1.84^q^	287.51±3.23^n-p^	54.67±0.27^l-o^
	2	35.40±0.15^b-d^	78.53±0.66^kl^	140.86±1.57^g-i^	250.99±2.77^gh^	54.36±0.23^k-n^
	3	34.43±0.18^a^	71.51±0.80^fg^	127.07±1.91^de^	248.72±3.34^fg^	51.33±0.28^d-f^
	4	35.49±0.18^c-e^	71.89±0.81^f-h^	150.92±1.91^k-l^	257.09±3.49^g-i^	49.79±0.29^bc^
	5	36.73±0.16^g-j^	66.15±0.72^de^	111.16±1.68^a^	193.23±3.02^a^	46.51±0.25^a^
3	1	-	87.46±0.59^o^	194.84±1.44^s^	272.11±2.59^j-k^	53.47±0.22^i-k^
	2	-	114.75±0.81^r^	181.50±1.99^q^	234.00±3.53^e^	50.8±0.30^de^
	3	-	80.25±0.84^l^	160.55±1.98^m^	323.45±3.41^tu^	-
	4	-	107.66±0.64^q^	165.36±1.55^m-o^	286.01±2.67^m-p^	56.66±0.23^r-t^
	5	-	66.86±0.60^e^	192.16±1.42^rs^	295.78±2.72^o-q^	53.78±0.21^j-l^
4	1	37.17±0.18^i-j^	87.82±0.77^o^	137.50±2.39^fg^	300.62±3.19^qr^	54.08±0.27^k-m^
	2	36.32±0.17^f-h^	81.13±0.77^lm^	153.13±1.80^l^	262.44±4.26^h-k^	52.1±0.37^f-h^
	3	36.20±0.14^fg^	63.53±0.68^cd^	144.29±1.62^g-k^	255.56±2.99^g-i^	51.39±0.25^d-f^
	4	37.09±0.14^i-j^	74.89±10.64^i^	132.63±1.55^ef^	259.16±2.71^g-i^	49.31±0.23^b^
	5	37.20±0.15^i-j^	64.46±0.69^de^	-	247.43±2.86^fg^	49.61±0.25^bc^
5	1	36.78±0.21^g-j^	84.12±0.93^n^	149.05±2.27^j-l^	324.86±3.74^tu^	56.35±0.32^q-s^
	2	37.45±0.26^j-k^	74.15±1.12^g-i^	168.88±2.63^n-p^	309.72±4.53^rs^	55.79±0.38^p-r^
	3	36.65±0.26^g-i^	78.36±0.63^j-l^	183.14±1.47^q^	327.84±2.52^uv^	58.07±0.21^uv^
	4	36.77±0.16^g-j^	79.58±0.68^kl^	172.12±1.61^op^	339.37±2.75^v^	58.43±0.23^vw^
	5	37.93±0.15^k-l^	81.27±0.66^lm^	172.64±1.61^p^	300.82±2.67^qr^	57.54±0.23^t-v^
6	1	36.56±0.19^g-i^	-	152.30±1.95^l^	286.85±3.36^m-p^	52.62±0.29^hi^
	2	35.71±0.16^d-f^	-	143.79±1.68^g-k^	263.95±2.90^i-l^	52.58±0.25^g-i^
	3	35.78±0.20^d-f^	-	147.51±2.12^i-l^	292.57±3.65^o-q^	56.88±0.31^st^
	4	36.10±0.20^e-g^	-	139.81±2.66^g-h^	274.63±4.51^k-m^	55.51±0.38^o-q^
	5	36.53±0.19^g-i^	-	138.32±1.97^f-h^	267.31±3.42^i-l^	54.77±0.29^l-p^
	6	34.71±0.31^ab^	-	130.26±3.30^de^	291.90±5.66^o-q^	55.47±0.48^o-q^
	7	34.73±0.22^ab^	-	164.37±2.32^mn^	230.34±4.10^c-e^	50.38±0.35^cd^
	8	34.76±0.29^ab^	-	113.35±3.03^ab^	249.75±5.23^k-h^	51.39±0.44^d-f^
7	1	-	95.50±0.77^p^	205.41±1.83^t^	380.21±3.17^w^	62.89±0.27^x^
	2	-	85.19±0.85^no^	184.91±1.20^q^	336.75±3.44^v^	59.29±0.29^w^
	3	-	74.63±0.85^hi^	187.15±2.03^qr^	315.26±3.51^st^	57.23±0.30^t-v^
	4	-	75.49±0.73^ij^	168.40±1.74^n-p^	284.38±3.00^m-o^	55.84±0.26^p-r^
	5	-	77.06±0.99^i-k^	181.26±2.37^q^	295.75±4.11+	54.9±0.35^m-p^
	6	-	70.33±0.76^f^	144.88±1.80^h-k^	297.82±3.15^p-r^	55.39±0.27^n-q^
	7	-	78.41±0.61^j-l^	147.26±1.43^i-l^	275.60±2.51^l-n^	55.02±0.22^m-p^

Means with same superscripts do not differ significantly (P<0.05)

^1^Means with three and more superscripts are written using ‘-’ between first and last

The LSM for ASM, BW20, BW40, EW28, EW40, EP40 and EM40 are presented in Table 4. All the production traits were significantly (P≤0.01) influenced by the generation, hatch and their interaction. The average LSM for ASM, EP40 and EM40 were 168.82±0.25 d, 72.60±0.41 eggs and 4.21±0.07 kg, respectively, in PD-3 line. The ASM showed decreasing trend, whereas egg production and egg mass showed an increasing trend over the generations. The performance of various traits was higher in early hatches compared to later hatches (Table 4). The LSM for interaction effects for production traits are presented in Table 5. The performance of different traits was higher in the later generations in early hatches in general with few exceptions.

**Table 4:** Least squares means of production traits in PD-3 line.

Particulars	ASM, d	Body weight, g		Egg prodn, no	Egg weight, g		Egg mass, kg
		20 wks	40 wks	40 wks	28 wks	40 wks	40 wks
**Overall LSM**	168.82±0.25 (4273)^1^	1304.53±3.02 (4070)	1682.93±4.42 (3227)	72.60±0.41 (4288)	49.40±0.08 (3477)	53.68±0.09 (3299)	4.21±0.02 (3296)
**Generation**	**	**	**	**	**	**	**
1	172.83±0.73^d^ (457)	1243.33±8.50^a^ (458)	1666.22±12.65^b^ (359)	72.20±1.19^e^ (456)	48.24±0.24^a^ (310)	53.65±0.26^bc^ (320)	4.19±0.07^b^ (320)
2	168.13±0.65^c^ (585)	1302.59±7.40^c^ (613)	1684.25±1.38^b^ (512)	65.78±1.07^b^ (576)	49.29±0.23^cd^ (450)	53.39±0.22^b^ (467)	3.85±0.06^b^ (467)
3	161.16±0.76^b^ (460)	1359.03±8.72^d^ (474)	1667.23±12.71^ab^ (351)	69.93±1.24^bc^ (462)	48.53±0.22^ab^ (400)	52.01±0.27^a^ (309)	4.10±0.07^b^ (309)
4	171.10±0.74^d^ (490)	1274.11±8.67^b^ (497)	1662.86±12.18^a^ (444)	78.14±1.258^d^ (481)	49.20±0.22^c^ (405)	53.46±0.25^b^ (410)	4.42±0.06^c^ (409)
5	174.72±0.68^d^ (473)	1234.74±7.80^a^ (494)	1655.89±10.78^a^ (441)	64.53±1.13^a^ (473)	48.86±0.21^b^ (364)	54.11±0.24^c^ (343)	3.83±0.06^a^ (343)
6	173.03±0.48^d^ (1159)	1328.40±6.62^c^ (846)	1733.88±7.45^c^ (1120)	69.06±0.79^b^ (1156)	50.17±0.15^d^ (749)	55.20±0.16^d^ (924)	4.05±0.04^b^ (922)
7	156.61±0.60^a^ (649)	1412.96±6.75^e^ (688)	**-**	90.88±0.95^e^ (684)	51.27±0.16^e^ (599)	53.09±0.20^b^ (526)	5.17±0.05^d^ (526)
**Hatch**	**	**	NS	**	**	**	**
1	163.19±0.49^a^ (961)	1350.95±5.68^c^ (967)	1672.69±8.77 (702)	78.90±0.80^c^ (960)	49.15±0.14^ab^ (861)	54.14±0.18^b^ (676)	4.67±0.05^e^ (676)
2	167.51±0.53^b^ (989)	1304.69±6.19^bc^ (1000)	1680.51±9.90 (706)	73.95±0.88^b^ (981)	48.74±0.16^a^ (798)	53.58±0.19^ab^ (738)	4.35±0.05^d^ (737)
3	168.60±0.56^bc^ (778)	1305.97±6.44^b^ (799)	1681.72±10.18 (572)	73.89±0.92^b^ (766)	49.66±0.16^b^ (651)	52.87±0.19^ab^ (601)	4.24±0.05^cd^ (600)
4	168.84±0.65^c^ (548)	1312.38±7.49^bc^ (575)	1650.94±11.42 (429)	67.48±1.06^ab^ (562)	49.54±0.21^b^ (443)	53.06±0.22^a^ (436)	3.82±0.06^ab^ (436)
5	169.81±0.67^c^ (598)	1305.37±7.72^bc^ (635)	1690.09±11.93 (467)	71.23±1.10^ab^ (618)	49.78±0.22^bc^ (440)	53.91±0.23^ab^ (490)	4.14±0.06^bc^ (489)
6	179.09±0.28^d^ (147)	908.78±24.23^a^ (51)	1730.14±22.73 (99)	68.22±2.11^a^ (149)	51.01±0.36^c^ (136)	55.16±0.42^c^ (132)	3.89±0.11^a^ (132)
7	175.71±1.29^d^ (177)	1303.09±26.39^b^ (43)	1735.88±20.16 (176)	68.49±2.13^a^ (178)	48.57±0.45^a^ (99)	54.41±0.45^c^ (157)	4.06±0.12^a^ (157)
8	178.09±1.70^d^ (75)	**-**	1761.89±25.94 (76)	72.57±2.83^b^ (74)	49.13±0.56^a^ (49)	55.32±0.54^c^ (69)	4.09±0.14^abc^ (69)
Gen x Hatch	**	**	**	**	**	**	**

^1^Values in the parentheses are number of observations. Means with same superscripts do not differ significantly

* (P<0.*05)*

** (P<0.01), NS = Non Significant

**Table 5:** Least squares means of interaction effects of production traits in PD-3 line.

Gen.	Hatch	ASM, d	Body weight, g		Egg prodn, no	Egg weight, g		Egg mass, kg
			20 wks	40 wks	40 wks	28 wks	40 wks	40 wks
1	1	157.56±1.39b-d^1^	1266.19±16.28f-i	1714.95±22.50e-j	88.65±2.29op	47.97±0.39b-d	55.17±0.58i-n	5.12±0.15q-s
	2	173.33±2.06n-r	1220.02±24.23e-g	1650.95±35.32a-g	77.33±3.41i-m	48.00±0.60b-d	52.57±0.73b-f	4.64±0.19m-p
	3	182.83±1.86v	1313.63±21.80i-k	1588.47±30.49ab	67.53±3.09b-h	47.38±0.56a-c	53.52±0.65d-i	3.79±0.17c-f
	4	174.50±2.04o-r	1324.71±24.00i-l	1572.61±34.09a	64.42±3.37b-f	46.62±0.63ab	53.08±0.70c-h	3.52±0.18bc
	5	172.33±1.60m-q	1366.88±18.77k-o	1719.32±25.77f-j	66.20±2.64b-g	-	54.09±0.54f-l	3.95±0.14c-i
	6	177.96±2.08r-v	908.78±24.23a	-	69.38±3.44c-j	51.08±0.58j-l	54.08±0.70e-l	3.92±0.18c-h
	7	171.30±2.25l-q	1303.09±26.39h-i	1751.02±35.32i-k	71.88±3.71e-k	48.41±0.76c-e	53.06±0.81c-h	4.36±0.21h-o
2	1	163.85±1.29e-h	1295.86±14.79h-i	1638.26±21.66a-f	75.54±2.13h-l	48.71±0.37c-f	53.32±0.46d-i	4.56±0.12k-o
	2	165.01±1.10f-i	1395.63±12.69m-p	1768.51±18.05j-k	75.48±1.83h-l	50.70±0.31g-l	53.90±0.37e-l	4.45±0.09j-o
	3	160.80±1.34d-f	1412.52±15.30n-p	1724.77±23.84f-j	70.97±2.25e-j	49.59±0.40d-j	53.80±0.49e-k	4.59±0.12l-o
	4	175.36±1.81o-s	1210.74±20.83d-f	1641.97±27.22a-g	44.91±2.99a	48.14±0.79b-d	52.81±0.59b-g	2.36±0.15a
	5	175.61±1.56p-t	1198.20±17.95c-e	1647.74±24.25a-g	62.01±2.61b-d	49.31±0.53d-h	53.10±0.48c-h	3.30±0.12b
3	1	161.68±1.28d-g	1418.45±15.06o-p	1688.54±22.73d-i	75.21±2.12h-l	50.99±0.35i-l	52.86±0.49d-g	4.57±0.12l-o
	2	156.74±1.23b-d	1335.94±14.37j-m	1674.88±23.20b-i	74.55±2.03j-l	46.05±0.36a	51.28±0.45bc	4.27±0.11g-n
	3	158.31±1.67b-d	1356.95±19.11j-n	1656.19±29.95a-h	68.93±2.79c-i	48.08±0.48b-d	48.76±0.63a	3.95±0.16c-i
	4	165.38±1.86f-j	1273.37±20.54g-i	1593.11±29.95a-c	59.96±2.97b	48.71±0.55c-f	51.17±0.67b	3.57±0.17bc
	5	163.7±2.22e-h	1410.45±26.09n-p	1723.42±34.49f-j	70.98±3.67e-j	48.84±0.67c-f	55.97±0.76mn	4.16±0.19f-l
4	1	169.19±1.37i-n	1314.83±16.00i-k	1627.95±21.46a-e	74.45±2.25j-l	49.30±0.39b-h	55.31±0.46j-n	4.40±0.12i-o
	2	175.78±1.48p-t	1338.41±17.14j-m	1698.64±24.53d-i	70.85±2.47d-j	46.67±0.45ab	53.86±0.48e-k	4.11±0.12e-k
	3	167.81±1.36h-m	1198.68±16.00c-e	1679.47±21.66c-i	84.49±2.30m-o	50.18±0.40f-k	53.01±0.44c-h	4.60±0.11l-p
	4	172.39±1.33m-q	1298.74±15.48h-i	1658.07±21.66a-h	80.77±2.21l-n	50.82±0.39h-l	52.19±0.44b-e	4.28±0.11g-n
	5	170.33±2.46i-o	1219.86±28.84e-g	1650.17±41.29a-g	80.15±4.17k-n	49.01±0.73d-f	52.94±0.87b-h	4.70±0.22n-q
5	1	170.54±1.44k-p	1356.52±16.73j-n	1659.21±23.33a-h	64.74±2.39b-f	46.63±0.43ab	54.61±0.53g-n	3.85±0.13c-g
	2	175.78±1.57p-t	1156.08±18.24b-d	1643.71±24.53a-g	58.94±2.58b	49.61±0.53d-j	54.36±0.59f-m	3.61±0.15b-d
	3	175.91±1.53q-t	1153.20±17.48bc	1622.27±23.71a-d	68.68±2.51c-i	50.04±0.45e-k	53.80±0.49e-k	3.92±0.12c-h
	4	170.59±1.50k-p	1392.20±17.39m-p	1697.67±23.84d-i	68.76±2.48c-i	49.14±0.46d-g	55.05±0.51i-n	4.08±0.13d-j
	5	180.79±1.55tv	1115.71±17.31b	1656.63±25.13a-h	61.53±2.56bc	48.90±0.46c-f	52.74±0.59b-g	3.68±0.15b-e
6	1	166.08±1.04g-k	1423.67±12.36o-p	1707.28±16.49d-i	77.91±1.72j-m	49.85±0.29e-k	54.19±0.36f-m	4.59±0.09l-o
	2	166.89±0.90h-l	1305.05±10.71h-i	1646.35±14.51a-g	70.52±1.51d-j	49.42±0.28d-i	54.79±0.33h-n	4.23±0.08f-m
	3	168.56±1.12h-n	1337.27±13.01j-m	1819.16±17.34k	70.10±1.85c-j	51.07±0.32k-l	54.58±0.38g-n	4.05±0.10d-j
	4	168.56±1.81h-n	1251.55±21.30e-h	1742.20±29.20h-k	63.04±2.97b-e	51.45±0.51kl	55.26±0.62j-n	3.89±0.16c-h
	5	175.74±1.20p-t	1324.45±14.32i-l	1743.30±18.53h-k	66.17±1.99b-g	50.79±0.35h-l	55.43±0.41k-n	3.93±0.10c-i
	6	180.22±1.50s-v	-	1730.14±22.73g-j	67.06±2.44b-h	50.93±0.42i-l	56.24±0.46n	3.85±0.12c-g
	7	180.12±1.27s-v	-	1720.73±19.46f-j	65.10±2.09b-f	48.73±0.47c-f	55.75±0.39l-n	3.76±0.10c-f
	8	178.09±1.70r-v	-	1761.89±25.94i-k	72.57±2.83f-l	49.13±0.56d-g	55.32±0.53j-n	4.09±0.14e-j
7	1	153.46±1.15ab	1381.16±13.47l-p	-	95.80±1.89p	50.63±0.33g-l	53.53±0.39d-j	5.61±0.10t
	2	159.05±1.15c-e	1381.72±13.47l-p	-	89.96±1.89o-p	50.73±0.33g-l	54.29±0.38f-n	5.14±0.10rs
	3	165.29±1.27f-j	1369.55±14.95k-o	-	86.51±2.12no	51.29±0.37kl	52.61±0.45b-f	4.80±0.11o-r
	4	155.10±1.63bc	1435.34±17.95p	-	90.53±2.52op	51.90±0.42l	51.87±0.55b-d	5.03±0.14p-s
	5	150.15±1.45a	1502.04±15.12q	-	91.59±2.14op	51.80±0.37l	53.12±0.44c-h	5.25±0.11st

Means with same superscripts do not differ significantly (P<0.*05)*

^1^Means with three and more superscripts are written using ‘-’ between first and last

### (co)Variance components

The appropriate model for different economic traits was selected based on the AIC values obtained in WOMBAT [21]. The AIC values of models for traits analyzed are presented in Table 6. The estimates of (co) variance components with respect to additive, maternal permanent environmental and residual effects arrived by employing the best model for growth traits are presented in Table 7. Model 5 was the best for BW0, BW2 and BW4 and model 4 for BW6 and SL6. The estimates of various models are presented in S1 Table in S1 Material. The variance was partitioned into additive direct, maternal genetic, maternal permanent environmental and residual variance for growth traits up to 4 weeks of age. BW6 and SL6 had all the effects, except maternal genetic variance. The appropriate models for production traits were model 4 for ASM, EP40 and EM40; model 1 for EW28 and EW40; model 5 for BW20 and model 2 for BW40, respectively (Table 8). The estimates of various models are presented in S2 Table in S1 Material. The ASM, EP40 and EM40 had additive direct, maternal permanent environmental and residual variance components, respectively. BW20 had the source of variation from additive direct, maternal genetic, maternal permanent environmental and residual components, while BW40 had similar sources, except for maternal permanent environmental variance. EW28 and EW40 had only additive genetic and residual components in Model 1.

**Table 6:** Comparison of AIC values from different models for growth and production traits of PD-3 line.

Trait	Model-1	Model-2	Model-3	Model-4	Model-5	Model-6
**BW0**	33949.08	32805.62	32510.55	32548.25	**32496.59**	32498.58
**BW2**	82912.93	82832.64	82834.62	82820.62	**82816.86**	82818.45
**BW4**	116384.18	116326.02	116326.76	116307.61	**116306.41**	116308.75
**BW6**	132867.48	132793.78	132795.58	**132773.29**	132773.38	132773.83
**SL6**	61038.47	60994.86	60996.42	**60980.52**	60980.79	60981.36
**ASM**	27010.35	27011.33	27009.65	**27006.60**	27008.60	27006.88
**BW20**	45463.89	45455.75	45457.19	45456.14	**45455.65**	45457.48
**BW40**	37580.22	**37567.69**	37569.31	37572.96	37569.68	37571.25
**EW28**	**12751.67**	12753.55	12755.14	12753.01	12755.02	12756.71
**EW40**	**12920.63**	12922.62	12924.47	12922.10	12924.10	12926.06
**EP40**	31414.33	31413.68	31414.58	**31405.83**	31407.83	31409.42
**EM40**	4177.00	4175.95	4177.92	**4173.43**	4175.25	4177.25

BW0: Day old body weight, BW2: 2^nd^ week body weight, BW4: 4^th^ week body weight, BW6: 6^th^ week body weight, SL6: 6^th^ week shank length, BW20: 20^th^ week body weight, BW40: 40^th^ week body weight, ASM: Age at sexual maturity, EP40: 40 weeks egg production, EM40: Egg mass upto 40 week, EW28: 28^th^ week egg weight, EW40: 40^th^ week egg weight, AIC, Akaike’s Information Criterion for the model obtained from WOMBAT

Column in bold represents estimates from best model as per AIC.

**Table 7:** Estimates of (co)variance components and genetic parameters for growth traits in PD-3 line.

Items^1^	BW 0	BW2	BW4	BW6	SL6
	Model-5	Model-5	Model-5	Model-4	Model-4
**σ**^**2**^_**a**_	0.25±0.48	39.58±7.81	165.05±31.79	786.52±115.82	5.43±0.79
**σ**^**2**^_**m**_	4.94±0.98	7.90±4.02	22.43±14.43	-	-
**σ**_**am**_	-	-	-	-	-
**σ**^**2**^_**c**_	2.32±0.60	13.64±3.25	58.57±12.79	253.17±34.49	1.40±0.23
**σ**^**2**^_**e**_	6.59±0.26	189.39±4.67	1127.77±22.46	3042.26±69.82	22.56±0.49
**σ** ^**2**^_**p**_	14.11±0.58	250.51±4.35	1373.82±19.99	4081.94±65.90	29.39±0.46
**h** ^**2**^	0.02±0.03	0.16±0.03	0.12±0.02	0.19±0.03	0.19±0.03
**m**^**2**^	0.35±0.06	0.03±0.02	0.02±0.01	-	-
**r**_**am**_	-	-	-	-	-
**c**^**2**^	0.17±0.05	0.05±0.01	0.04±0.01	0.06±0.01	0.05±0.01
**h**^**2**^_**T**_	0.19	0.17	0.13	0.19	0.19

^1^σ^2^_a,_ σ^2^_c,_ σ^2^_m,_ σ^2^_e_ and σ^2^_p_ are additive direct, maternal permanent environmental, maternal genetic, residual variance and phenotypic variance, respectively; h^2^, heritability; m^2^, maternal heritability; r_am,_ direct-maternal genetic correlation; c^2^ = σ^2^_c_/σ^2^_p_; h^2^_t_ is total heritability

**Table 8:** Estimates of (co)variance components and genetic parameters for production traits of PD-3 line.

Items^1^	ASM	BW20	BW40	EW28	EW40	EP40	EM40
	Model-4	Model-5	Model-2	Model-1	Model-1	Model-4	Model-4
**σ**^**2**^_**a**_	42.14±9.07	4861.83±1244.07	13091.30±3029.05	7.01±0.83	6.92±0.96	64.01±19.65	0.15±0.05
**σ**^**2**^_**m**_	-	872.47±640.70	4277.19±1326.84	-	-	-	-
**σ**_**am**_	-	-	-	-	-	-	-
**σ**^**2**^_**c**_	7.57±3.26	808.87±563.58	-	-	-	27.35±8.95	0.05±0.02
**σ**^**2**^_**e**_	174.27±6.41	23773.00±884.24	35434.20±1866.44	9.58±0.54	13.61±0.68	511.19±16.33	1.09±0.04
**σ** ^**2**^_**p**_	223.97±5.60	30316.20±769.20	52802.70±1665.04	16.59±0.52	20.53±0.62	602.56±14.14	1.30±0.03
**h** ^**2**^	0.19±0.04	0.16±0.04	0.25±0.05	0.42±0.04	0.34±0.04	0.11±0.03	0.12±0.04
**m**^**2**^	-	0.03±0.02	0.08±0.02	-	-	-	-
**r**_**am**_	-	-	-	-	-	-	-
**c**^**2**^	0.03±0.01	0.03±0.02	-	-	-	0.05±0.02	0.04±0.02
**h**^**2**^_**T**_	0.19	0.17	0.29	0.42	0.34	0.11	0.12

^1^σ^2^_a,_ σ^2^_c,_ σ^2^_m,_ σ^2^_e_ and σ^2^_p_ are additive direct, maternal permanent environmental, maternal genetic, residual variance and phenotypic variance, respectively; h^2^, heritability; m^2^, maternal heritability; r_am,_ direct-maternal genetic correlation; c^2^ = σ^2^_c_/σ^2^_p_; h^2^_t_ is total heritability

### Heritability

**Fig 1:**
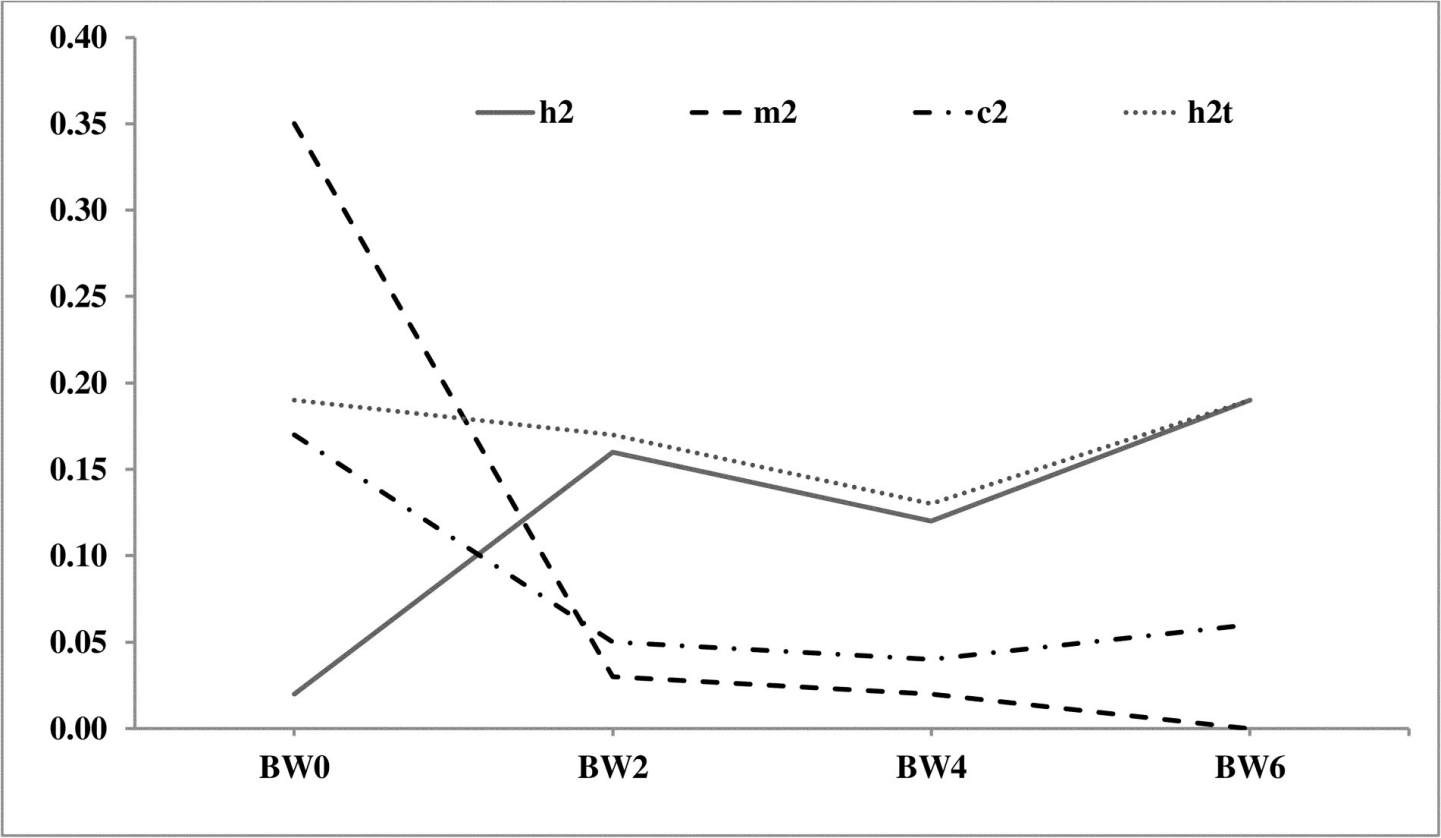
Estimates of heritability (h^2^), maternal heritability (m^2^), proportion of maternal permanent environmental variance on phenotypic variance (c^2^) and total heritability (h^2^_t_) for growth traits. BW0: Day old body weight, BW2: 2^nd^ week body weight, BW4: 4^th^ week body weight, BW6: 6^th^ week body weight.

**Fig 2:**
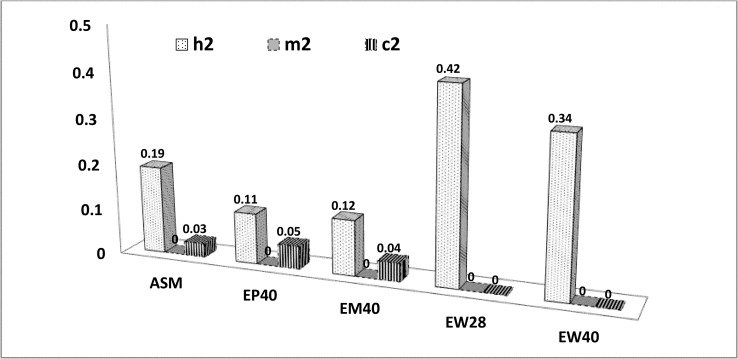
Estimates of heritability (h^2^), maternal heritability (m^2^) and proportion of maternal permanent environmental variance on phenotypic variance (c^2^) for production traits. ASM: Age at sexual maturity, EP40: 40 weeks egg production, EM40: Egg mass up to 40 weeks, EW28: 28^th^ week egg weight, EW40: 40^th^ week egg weight.

The heritability (h^2^) estimates were low to moderate in magnitude for all the growth traits and ranged from 0.02±0.03 to 0.19 ±0.03 (Table 7). The trend of additive and maternal heritability estimates for growth and production traits over the age was depicted in Figs 1 and 2, respectively. The maternal heritability (m^2^) at birth was high in magnitude (0.35±0.06), which reduced gradually by the end of the 4^th^ week (Fig 1). The c^2^ was also higher at birth and gradually reduced with age. The ASM was moderately heritable (0.19±0.04). The heritability of EP40 and EM40 was low with 0.11±0.03 and 0.12±0.04 h^2^ estimates, respectively. BW20 and BW40 had very less magnitude of maternal h^2^, while ASM, BW20, EP40 and EM40 had low magnitude c^2^. The heritability was high for egg weight (EW28 and EW40) and moderate for adult body weight (EW20 and EW40) in PD-3 line (Table 8, Fig 2).

### Correlations

The direct additive genetic correlation (r_a_) between BW0 and other juvenile traits was less in magnitude and positive, while maternal genetic (r_g_) and maternal permanent environmental (r_c_) correlation were significantly higher; phenotypic correlation (r_p_) was less in magnitude (Table 9). The r_g_ was observed till 4 weeks of age and r_c_ continued up to 20 weeks of age. The r_a_ between BW2, BW4, BW6 and SL6 was highly correlated with significant (P≤ 0.05) positive association. The r_c_ was observed up to 20 weeks of age with significant high correlation coefficients between the body weights and shank length. The r_p_ was higher among BW2, BW4, BW6 and SL6 with a positive association. The r_a_ between BW6 and adult body weights (BW20 and BW40) was high in magnitude.

**Table 9:** Correlations among different growth traits in PD-3 line.

Trait combinations	Direct additive genetic correlation (r_a_)	Maternal genetic correlation (r_m_)	Maternal permanent environmental correlation (r_c_)	Phenotypic correlation (r_p_)	Number of observations
**BW0 and BW2**	0.45±0.34^NS^	0.96±0.11**	0.76±0.11**	0.27±0.02**	7362
**BW0 and BW4**	0.18±0.32^NS^	0.74±0.22**	0.43±0.15**	0.13±0.02**	8853
**BW0 and BW6**	0.28±0.29^NS^	-	0.57±0.11**	0.12±0.02**	9326
**BW0 and SL6**	0.28±0.29^NS^	-	0.51±0.12**	0.12±0.02**	9306
**BW2 and BW4**	0.81±0.08**	0.79±0.21*	0.91±0.05**	0.64±0.01**	11750
**BW2 and BW6**	0.69±0.07**	-	0.87±0.05**	0.56±0.01**	11643
**BW2 and SL6**	0.73±0.06**	-	0.91±0.05**	0.52±0.01**	11432
**BW4 and BW6**	0.98±0.02**	-	0.92±0.03**	0.64±0.01**	13257
**BW4 and SL6**	0.95±0.02**	-	0.87±0.05**	0.56±0.01**	13055
**BW6 and SL6**	0.93±0.02**	-	0.94±0.02**	0.84±0.01**	13992
**BW6 and BW20**	0.61±0.09**	-	0.33±0.21^NS^	0.30±0.02**	3905
**BW6 and BW40**	0.50±0.10**	-	-	0.12±0.02**	3062

* P≤0.05

** P≤0.01; NS, Non significant.

The correlation coefficients from different components between production traits are presented in Table 10. The r_a_ between ASM and BW20 was negative as heavier birds matured early. ASM and BW40 had a low degree of positive association. ASM had significantly (P≤ 0.05) higher negative correlation (r_a_, r_c_ and r_p_) with EP40 and EM40 (Table 10). The r_a_ and r_p_ between ASM and egg weights (EW28 and EW40) were of low magnitude and positive in direction. The r_a_ between EP40 and BW40 was negative, while between EP40 and BW20 was positive with less magnitude. EP40 and EM40 had highly significant (P≤ 0.05) positive association with r_a_, r_c_ and r_p_ with. The EP40 and egg weight (EW28 and EW40) had negative association, while EM40 and egg weight had a positive association for both r_a_ and r_p_. The EM40 had a positive association with all the production traits, except ASM. The adult body weight (BW20 and BW40) and egg weight (EW28 and EW40) had highly significant (P≤ 0.05) positive r_a_ and low magnitude r_p._ The BW20 and BW40 had highly significant (p≤ 0.05) positive association for r_a_ and r_g_ and r_p_ components.

**Table 10:** Correlation coefficients among different production traits in PD-3 line.

Trait combinations	Direct additive genetic correlation (r_a_)	Maternal genetic correlation (r_m_)	Maternal permanent environmental correlation (r_c_)	Phenotypic correlation (r_p_)	Number of observations
**ASM and BW20**	-0.30±0.15^NS^	-	-0.57±0.33^NS^	-0.23±0.02**	3936
**ASM and BW40**	0.29±0.14^NS^	-	-	0.03±0.02^NS^	3183
**ASM and EP40**	-0.94±0.06**	-	-0.51±0.21^NS^	-0.50±0.01**	4215
**ASM and EM40**	-0.90±0.06**	-	-0.43±0.28^NS^	-0.56±0.01**	3242
**ASM and EW28**	0.24±0.10*	-	-	0.09±0.02**	3433
**ASM and EW40**	0.29±0.11*	-	-	0.11±0.02**	3244
**EP40 and BW20**	0.14±0.19^NS^	-	0.49±0.31^NS^	0.21±0.02**	3946
**EP40 and BW40**	-0.31±0.16^NS^	-	-	0.01±0.02^NS^	3177
**EP40 and EM40**	0.89±0.04**	-	0.98±0.01**	0.96±0.01**	3287
**EP40 and EW28**	-0.15±0.13^NS^	-	-	0.01±0.02^NS^	3456
**EP40 and EW40**	-0.28±0.14^NS^	-	-	-0.07±0.02*	3287
**EM40 and BW20**	0.48±0.16*	-	0.41±0.35^NS^	0.29±0.02**	2979
**EM40 and BW40**	0.02±0.17^NS^	-	-	0.10±0.02**	2681
**EM40 and EW28**	0.26±0.13^NS^	-	-	0.21±0.02**	2708
**EM40 and EW40**	0.13±0.13^NS^	-	-	0.23±0.02**	3287
**EW28 and BW20**	0.64±0.08**	-	-	0.27±0.02**	3237
**EW28 and BW40**	0.69±0.07**	-	-	0.29±0.02**	2552
**EW28 and EW40**	0.98±0.02**	-	-	0.57±0.02**	2710
**EW40 and BW20**	0.70±0.07**	-	-	0.22±0.02**	2982
**EW40 and BW40**	0.74±0.07**	-	-	0.34±0.02**	2684
**BW20 and BW40**	0.97±0.05**	0.77±0.24*	-	0.39±0.02**	2888

* P≤0.05

** P≤0.01; NS, Non significant.

### Breeding value and genetic gain

**Fig 3:**
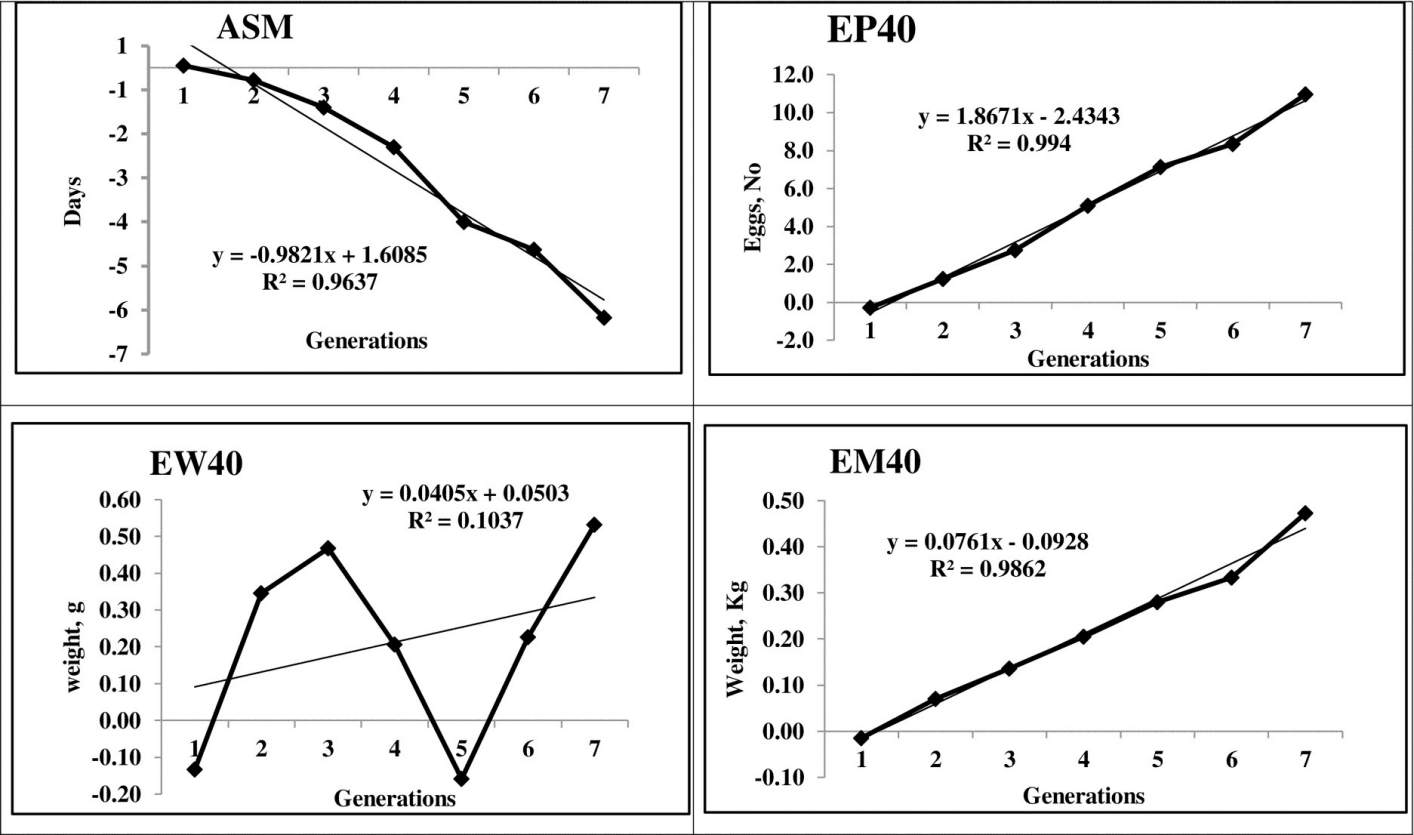
Trend of breeding value for important economic traits over generations. ASM: Age at sexual maturity, EP40: 40 weeks egg production, EM40: Egg mass up to 40 weeks, EW40: 40th week egg weight.

**Fig 4:**
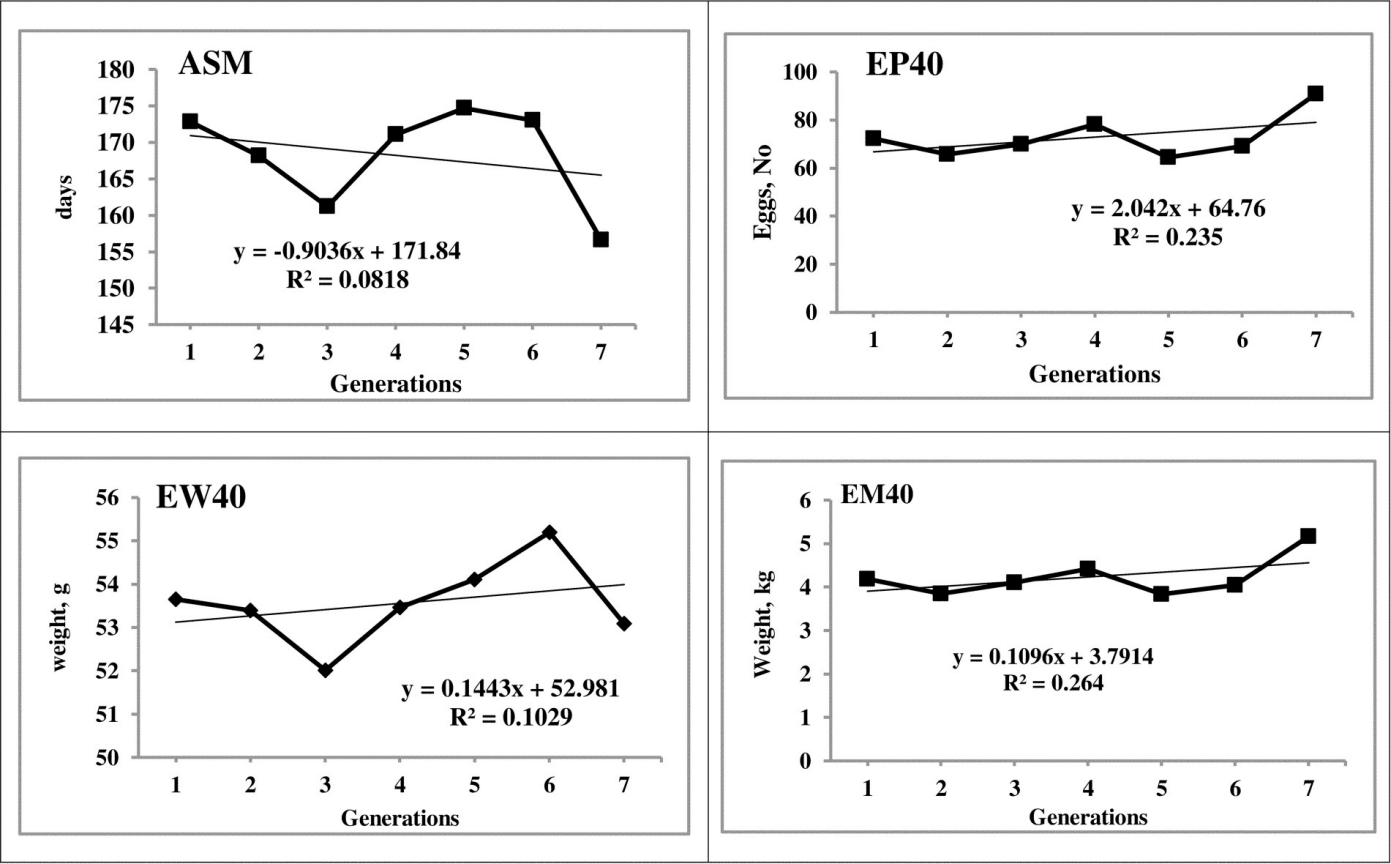
Phenotypic trend of important economic traits over generations. ASM: Age at sexual maturity, EP40: 40 weeks egg production, EM40: Egg mass up to 40 weeks, EW40: 40th week egg weight.

The average EBV for BW6 and SL6, the important juvenile traits, was 17.65 g and 1.33 mm, respectively during the S-7 generation. The genetic trend of BW6 and SL6 showed significant (P < 0.05) and linear increment over the generations. The EBV of EM40 (0.48 kg) at the end of seventh generation significantly (P≤ 0.05) increased linearly as a response to selection in PD-3 line. The average genetic and phenotypic response was 0.08 and 0.11 kg per generation for EM40, the primary trait of selection (Figs 3 and 4). ASM decreased significantly with an increment of one day in each generation. The breeding value of EP40 showed an increasing trend with a genetic gain of 1.87 eggs per generation. The EW28 and EW40 increased linearly with an average genetic gain of 0.09 and 0.04 g, respectively per generation.

### Inbreeding

**Fig 5:**
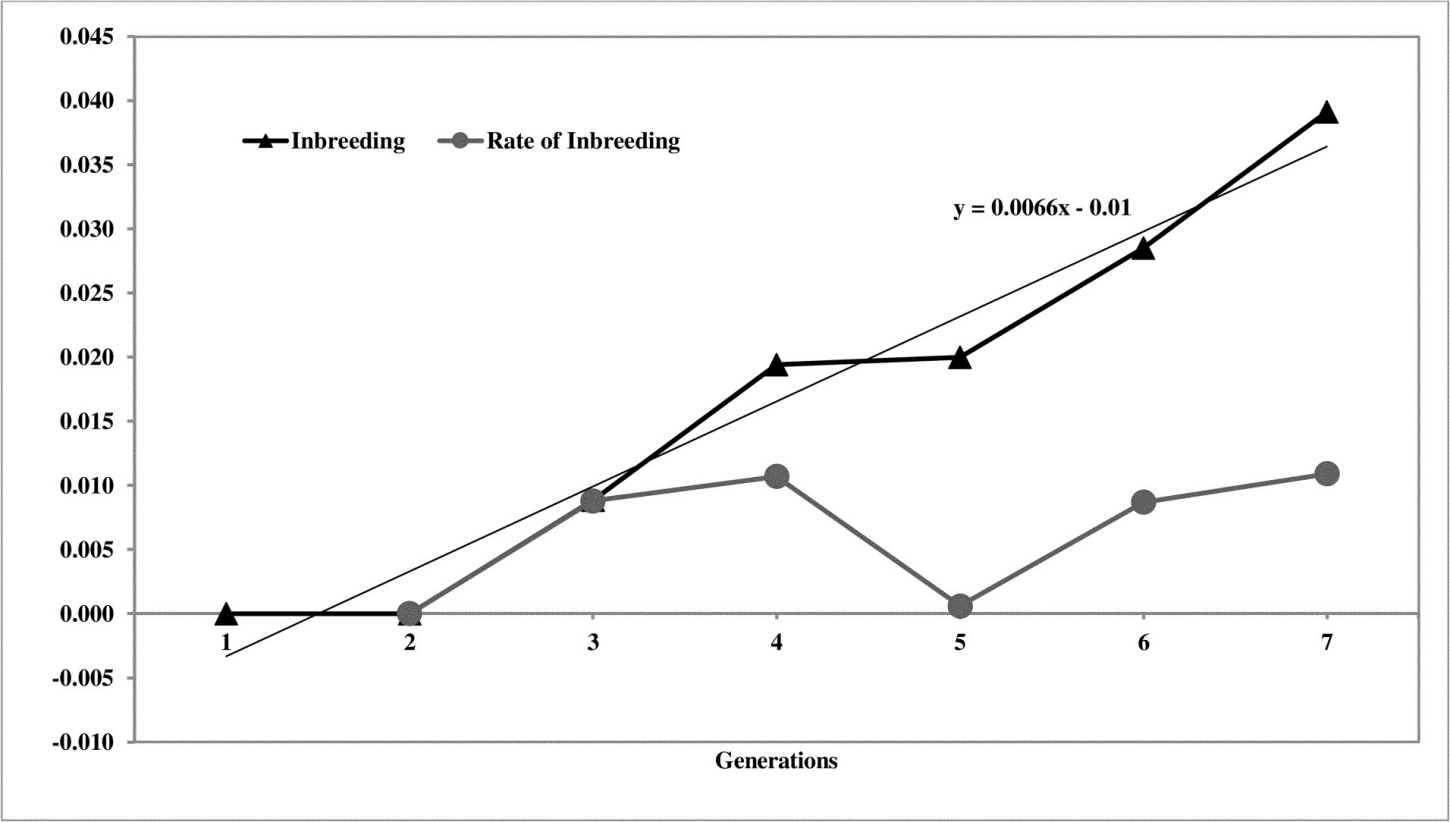
Status of inbreeding over the generations.

The inbreeding was almost negligible in the population. The average inbreeding coefficient of the population was 0.019 and the average ΔF was 0.007 over the last seven generations of selection. The inbreeding coefficient of the population increased gradually from 0.00 to 0.04 in the 7^th^ generation. The trend of inbreeding and rate of inbreeding is presented in Fig 5.

## Discussion

The effects of generation, hatch and their interaction were significant (P≤ 0.05) on body weight and shank length (Table 2) with an increasing linear trend over the generations and decreasing trend among the hatches. Significant effect of generation and hatch on body weight and shank length similar to the present findings was reported in PD-1 line [4, 12], in PB-2 line [17] and in White Leghorn layer strains [14]. The higher body weight in the early phase of life is important as it helps the bird to attain maturity at an early age and aid indirectly in increasing the egg production. Higher body weight in a female parent may contribute to the higher body weight in the terminal cross, which was desirable in a rural chicken variety. The LSM was significantly higher either in the first or second hatch, which may be due to the variations in environmental factors like maternal effects, hatching conditions and rolling reactions etc.

The LSM of ASM significantly (P≤ 0.01) reduced over the generations as a correlated response to the selection for EM40 in PD-3 line. The increased body weight over the generations may have resulted in reduced ASM in a positive direction. Selection for body weight resulted in the reduction of ASM though direct selection was not practiced for this trait [27]. The significant (P≤ 0.01) increase in adult body weight (BW20 and BW40) might be attributed to the management, environment and feed restriction schedule followed during the grower phase to maintain the target body weight at laying. The body weight and egg production had a negative relationship in PD-3 line in the present study. The negative association between body weight and egg production in chicken was documented by many authors [4, 7, 11, 20, 28]. Higher early body weight is of great importance for the onset of egg production as heavier birds mature early and lay more number of eggs. The egg production (EP40) has shown a significant increasing trend with fluctuations across the generations (Table 4) as a correlated response to selection for higher egg mass as both are positively correlated traits. The EW28 showed a gradually increasing trend, while EW40 showed a fluctuating trend with the egg weight stabilized between 53 and 55 g. Selection for higher egg mass might be the possible reason for maintaining egg weight without any reduction, in spite of the increased EP40 as selection based on EM40 takes care of both egg number and egg weight [20]. The EM40 showed an increasing trend with exceptions during 2^nd^ and 5^th^ generation where lower egg mass was observed, which was due to the reduced egg production in the generation. However, a significant positive trend was observed for EM40 over the generations (Table 4).

Model 5 was appropriate for juvenile body weight (BW0, BW2 and BW4) with a, m, c, r and p effects and Model 4 for BW6 and SL6 with a, c, r and p effects. Maternal effects, both m and c were observed up to 20 weeks of age in PD-3 (Table 8). Jasouri et al. (2017) observed maternal genetic effects on body weight up to 12 weeks of age in dual purpose chicken [29]. The reduction in maternal effects as age increased was reported in broiler crosses [9] and commercial broilers [30]. In the present study, the maternal genetic effect continued till 40 weeks of age, contrary to the earlier findings (Table 8). Maternal environment effects are pre and post-ovipositional, the later may be pre and post hatch effects which influence egg quality traits and chick weight at hatch [31]. Maternal effects are due to the contribution of maternal additive, dominant gene effects and the differences between favorable and unfavorable allele frequencies [9]. The maternal effects were observed on growth traits up to six weeks of age in PD-1 line [4] and PB-2 broiler female line [17], contrary to the present findings. The continued existence of maternal effects in PD-3 line beyond juvenile phase might be due to the breed characteristic as *Dahlem Red* breed is one of the excellent female parent lines. The inclusion of one of the maternal effects either m, or am or c in the model could be enough to adjust the variation that occurred due to all the maternal effects [32]. The importance of “m” effects for early body weight was demonstrated, though the magnitude was less than that of “c” effects [7, 8] in different chicken populations.

Model 4 was the appropriate model for ASM, EP40 and EM40 with effects of a and c, while model 1 was the best for EW28 and EW40 with direct additive genetic effect. Similar to the present findings, maternal effects in addition to the direct additive effect for the egg production and egg weight [29, 33, 34] were reported in chicken. However, some authors reported that model 1 without or with negligible maternal effects was suitable for egg production traits [8, 35, 36], contrary to the present study. A comprehensive review of the literature revealed that additive genetic effect increased and maternal effects decreased with age for production traits [37–39].

The estimates of h^2^ determined using the animal model was more precise due to partitioning of the variance and covariance into all possible sources. The h^2^ estimates of low magnitude with high precision and accuracy using the animal model were documented in the literature [5, 7, 8, 29]. The variation in h^2^ estimates over the time was due to the activation of various genes responsible for egg production [39] and persistency [40]. Higher h^2^ estimates for body weight (0.24–0.47), ASM (0.36), egg number (0.17), egg mass (0.16) and egg weight (0.32–0.43) were observed in Mazandaran chicken [39] due to ignoring the maternal effects compared to the present estimates. The lower estimates in the present study might be due to the prolonged selection for higher egg mass, which reduced the variability in the population and also due to the inclusion of additional sources of variance in the model. Higher magnitude of h^2^ estimates was also reported for egg production in Horro chicken from Ethiopia [41] in commercial layer line [42] and in Korean chicken [43].

The heritability estimated using the animal model was lesser as compared to the Henderson variance component analysis model. Maternal effects account for only small portion (2–8%) of the variance in chicken, but ignoring these effects results in biasness and over estimation of genetic parameters [35, 36, 44] leading to inadequate inferences hampering the breeding goals [6, 34, 45]. Similar to the findings of the present study, h^2^ estimates for growth and egg production traits were reported in PD-1 [4]; in PB-2 [17] from India using the REML including the maternal effects in the model. The precise and accurate h^2^estimates in the present study were due to the inclusion of maternal effects in the model, which will aid the breeder in planning the breeding program with reliable prediction.

Genetic correlation plays a significant role in the success of the breeding experiment, as selection for one trait improves the performance in other traits as a positive correlated response, whereas negative association depresses the performance [7, 11]. In addition, r_a_, r_c_ continue to exist up to 20 weeks of age (Table 9) indicating the existence of prolonged maternal effects as PD-3 line is a female line, contrary to the findings in other lines wherein the maternal effects ceased at an early age [4, 17]. The factors like hatch conditions, egg size and uterus have considerable influence on early body weight leading to the high effect of c, which reduces or becomes negligible at later stages of life [4]. However, in this study, effect of c prolonged up to 40 weeks of age. The r_g_ between growth traits (Body weight and Shank length) was high in magnitude in PD-3 line in the present study, similar to the reports in PD-1 line [4] in PB-2 broiler line [17]; in Mazandaran chicken [39]; in Thai native chicken [46].

The correlation (r_a_, r_c_ and r_p_) between ASM and EP40 was significant with a high degree of negative association in PD-3 line. A similar significant inverse relationship between ASM and egg production in chicken was reported by many authors [4, 11, 17]. The correlation between ASM and egg weight was positive as the birds matured late laid heavier eggs. The association between ASM and BW20 was negative and was in the desirable direction as heavier birds matured earlier. A similar association between ASM and egg weight; and ASM and BW20 were reported by many authors [11, 14]. The association (r_a_) between BW40 and EP40 was negative (Table 10). The inverse relationship between body weight and egg production was an established fact in chicken [7, 11, 28]. The EM40 had a direct positive association with EP40, EW28 and EW40, while had an antagonistic relationship with ASM. The direct positive association was justified as egg mass is the product of egg number and egg weight, and the higher number of eggs with better egg weight resulted in higher egg mass. The selection for egg mass maintains both egg number and egg weight at an optimum level, which is commonly practiced in breeder lines [20]. The early matured bird recorded higher egg mass with an inverse relationship as these birds produced more number of eggs with optimum egg weight leading to higher egg mass. The direction, magnitude and precision of correlation enable the breeder to fix the favourable traits in selection for improvement of the traits simultaneously [4]. Multi trait selection with adequate importance to each trait is the better option for optimal growth and egg production in chicken as these traits have antagonistic relationships making it more complex.

The average EBV of EM40, the primary trait of selection recorded a significant linear trend over the generations indicating the positive selection response in the population with significant improvement (Fig 3). The positive genetic trend was observed in BW6 and SL6 as correlated responses to selection, though the selection was not practiced for growth traits in this line. Similar observations of indirect response in correlated traits were reported in other chicken lines [4, 17, 20]. The direct positive genetic trend for BW6 and SL6 was observed in PD-6, a rural male parent line [47]. The EBV of ASM reduced linearly over the generations as an indirect response to selection for egg mass. The EBV of EP 40 showed a linear increasing trend with an average genetic gain of 1.87 eggs per generation (Fig 3), while the phenotypic trend showed fluctuations (Fig 4). The positive genetic and phenotypic gains in the population might be due to the effect of selection for egg mass, which improved both egg production and egg weight. The EW40 showed a linear increasing trend with fluctuations over the generations as selection for egg mass maintained the egg weight in the population. The EBV and the genetic trend observed in all the production traits was due to the correlated response to selection for egg mass as the direct selection was not practiced for those traits.

The ΔF in the population was very low (0.007), which may be due to the ideal breeding strategy followed in the PD-3 line, wherein close relatives were not included in the mating plan. The number of sires and dams utilized and contributed was higher than those required for maintaining effective population size. The inbreeding coefficient of the population at the end of the 7^th^ generation was 0.019. The inbreeding was not set in PD-3 chicken or negligible after 7 generations of selection. The delayed onset of inbreeding in pedigreed breeding populations similar to the present study was reported in pureline chicken [20, 47]. The PD-3 population was in ideal condition with respect to genetic architecture after seven generations; however, the inbreeding coefficient may increase in further generations of selection.

## Conclusion

The EBV of primary and associated traits was positive with a linear trend in the desired direction indicating the efficacy of selection practiced in PD-3 line with negligible inbreeding over the seven generations. Partitioning of variance into additive, maternal genetic, maternal genetic permanent environmental and residual sources improved the precision of the BV and genetic parameters. The accuracy in EBV and the genetic parameters aids in selecting the suitable breeding strategy for genetic improvement of PD-3 line. The study concluded that the positive genetic gains with respect to economic traits in PD-3 line lead to improvement in terminal crosses intended for backyard poultry farming.

## Supporting information

S1 Material (PDF)
