# Supplementary material for: Analysis of (co) variance components and estimation of breeding value of growth and production traits in Dahlem Red chicken using pedigree relationship in an animal model
Source: PLoS One. 2021 Mar 3;16(3):e0247779. doi: 10.1371/journal.pone.0247779 (PMC7928508; doi:10.1371/journal.pone.0247779)
Supplement: S1 Material — (PDF) [file pone.0247779.s001.pdf]

S1 Table. Estimates of (co)variance components and genetic parameters for growth traits PD-3 line.

Items[+]	Model-1	Model-2	Model-3	Model-4	Model-5	Model-6
**TRAIT: BW0**						
σ^2_a	7.12	0.38	0.46	1.61	**0.25**	0.26
σ^2_m	-	8.99	9.09	-	**4.94**	4.96
σ_{am}	-	-	-0.18	-	**-**	-0.02
σ^2_c	-	-	-	5.90	**2.32**	2.32
σ^2_e	4.87	6.53	6.49	5.93	**6.59**	6.59
σ^2_p	11.98	15.90	15.87	13.44	**14.11**	14.11
h^2	0.59±0.02	0.02±0.03	0.03±0.04	0.12±0.04	**0.02±0.03**	0.02±0.04
m^2	-	0.57±0.02	0.57±0.04	-	**0.35±0.06**	0.35±0.07
r_{am}	-	-	-0.09±0.26	-	**-**	-0.02±0.48
c^2	-	-	-	0.44±0.02	**0.17±0.05**	0.16±0.05
h^2_T	0.59	0.31	0.30	0.12	0.19	0.19
**AIC**	33949.076	32805.622	32510.55	32548.252	**32496.586 (best)**	32498.584
**TRAIT: BW2**						
σ^2_a	97.37	45.39	45.72	44.43	**39.58**	41.49
σ^2_m	-	25.97	26.52	-	**7.90**	9.58
σ_{am}	-	-	-0.85	-	**-**	-3.39
σ^2_c	-	-	-	18.49	**13.64**	13.91
σ^2_e	165.72	187.08	186.91	187.06	**189.39**	188.44
σ^2_p	263.09	258.43	258.30	249.98	**250.51**	250.03
h^2	0.37±0.03	0.18±0.03	0.18±0.03	0.18±0.03	**0.16±0.03**	0.17±0.04
m^2	-	0.10±0.01	0.10±0.02	-	**0.03±0.02**	0.04±0.02
r_{am}	-	-	-0.02±0.16	-	**-**	-0.17±0.23
c^2	-	-	-	0.07±0.01	**0.05±0.01**	0.06±0.01
**h2T**	0.37	0.23	0.22	0.18	0.17	0.16
**AIC**	82912.928	82832.640	82834.622	82820.622	**82816.864 (best)**	82818.454
**TRAIT: BW4**						
σ^2_a	370.29	191.91	207.77	181.75	**165.05**	188.26
σ^2_m	-	92.57	110.78	-	**22.43**	36.57
σ_{am}	-	-	-30.62	-	**-**	-28.43
σ^2_c	-	-	-	71.83	**58.57**	58.82
σ^2_e	1050.75	1119.59	1111.70	1119.65	**1127.77**	1116.24
σ^2_p	1421.03	1404.07	1399.63	1373.23	**1373.82**	1371.47
h^2	0.26±0.02	0.14±0.02	0.15±0.03	0.13±0.02	**0.12±0.02**	0.14±0.03
m^2	-	0.07±0.01	0.08±0.02	-	**0.02±0.01**	0.03±0.01
r_{am}	-	-	-0.20±0.45	-	**-**	-0.341±0.19
c^2	-	-	-	0.05±0.01	**0.04±0.01**	0.04±0.01
h^2_T	0.26	0.17	0.16	0.13	0.13	0.12
**AIC**	116384.178	116326.02	116326.758	116307.608	**116306.408 (best)**	116308.748
**TRAIT: BW6**						
σ^2_a	1503.58	899.84	927.77	**786.52**	761.89	841.85
σ^2_m	-	330.94	348.32	**-**	62.67	93.26
σ_{am}	-	-	-37.21	**-**	-	-92.29
σ^2_c	-	-	-	**253.17**	212.26	219.36
σ^2_e	2771.77	2997.61	2985.68	**3042.26**	3053.88	3015.08
σ^2_p	4275.36	4228.39	4221.56	**4081.94**	4090.59	4077.25
h^2	0.35±0.02	0.21±0.03	0.22±0.03	**0.19±0.03**	0.19±0.03	0.21±0.03
m^2	-	0.08±0.01	0.08±0.01	**-**	0.02±0.01	0.02±0.02
r_{am}	-	-	-0.07±0.14	**-**	-	-0.33±0.21
c^2	-	-	-	**0.06±0.01**	0.05±0.01	0.05±0.01
h^2_T	0.35	0.25	0.25	0.19	0.19	0.18
**AIC**	132867.484	132793.776	132795.58	**132773.288 (best)**	132773.382	132773.83

TRAIT: SL6						
**σ^2_a**	9.36	6.06	6.35	**5.43**	5.28	5.87
**σ^2_m**	-	1.78	1.96	**-**	0.37	0.57
**σ_{am}**	-	-	-0.39	**-**	-	-0.60
**σ^2_c**	-	-	-	**1.40**	1.17	1.20
**σ^2_e**	21.08	22.32	22.18	**22.56**	22.63	22.34
**σ^2_p**	30.44	30.15	30.09	**29.39**	29.44	29.38
**h^2**	0.31±0.02	0.20±0.03	0.21±0.03	**0.19±0.03**	0.18±0.03	0.20±0.03
**m^2**	-	0.06±0.01	0.07±0.01	**-**	0.01±0.01	0.02±0.01
**r_{am}**	-	-	-0.11±0.15	**-**	-	-0.33±0.21
**c^2**	-	-	-	**0.05±0.01**	0.04±0.01	0.04±0.01
**h^2_T**	0.31	0.23	0.22	0.19	0.19	0.18
**AIC**	61038.466	60994.864	60996.42	**60980.52 (Best)**	60980.786	60981.356

Values in the parentheses are standard errors; Column in bold represents estimates from best model as per AIC
+σ^2_a, σ^2_c, σ^2_m, σ^2_e and σ^2_p are additive direct, maternal permanent environmental, maternal genetic, residual variance and phenotypic variance, respectively; h^2, heritability; m^2, maternal heritability; r_{am}, direct-maternal genetic correlation; $c^2 = \sigma^2_c/\sigma^2_p$; h^2_t is total heritability; AIC, Akaike's Information Criterion for the model obtained from WOMBAT

S2 Table. Estimates of (co)variance components and genetic parameters for production traits PD-3 line.

Items[+]	Model-1	Model-2	Model-3	Model-4	Model-5	Model-6
**TRAIT: ASM**						
σ^2_a	57.52	53.09	72.49	**42.14**	42.18	61.83
σ^2_m	-	2.83	7.64	**-**	0.001	3.62
σ_{am}	-	-	-14.21	**-**	-	-12.94
σ^2_c	-	-	-	**7.57**	7.56	7.72
σ^2_e	169.77	171.07	161.19	**174.27**	174.25	164.32
σ^2_p	227.29	226.99	227.13	**223.97**	223.99	224.55
h^2	0.25±0.03	0.23±0.04	0.32±0.06	**0.19±0.04**	0.19±0.04	0.28±0.06
m^2	-	0.01±0.01	0.03±0.02	**-**	0.00±0.01	0.02±0.02
r_{am}	-	-	-0.60±0.18	**-**	-	-0.87±0.34
c^2	-	-	-	**0.03±0.01**	0.03±0.02	0.03±0.02
h^2_T	0.25	0.24	0.24	0.19	0.19	0.20
AIC	27010.352	27011.334	27009.65	**27006.602 (best)**	27008.602	27006.884
**TRAIT: BW20**						
σ^2_a	7758.15	5196.00	6160.01	5569.33	**4861.83**	5421.56
σ^2_m	-	1538.71	1801.36	-	**872.47**	1048.44
σ_{am}	-	-	-743.29	-	**-**	-395.38
σ^2_c	-	-	-	1312.66	**808.87**	755.30
σ^2_e	22966.50	23788.10	23291.30	23438.40	**23773.00**	23496.60
σ^2_p	30724.60	30523.40	30509.30	30310.40	**30316.20**	30326.50
h^2	0.25±0.03	0.17±0.04	0.20±0.06	0.18±0.04	**0.16±0.04**	0.18±0.06
m^2	-	0.05±0.02	0.06±0.02	-	**0.03±0.02**	0.04±0.02
r_{am}	-	-	-0.22±0.23	-	**-**	-0.17±0.32
c^2	-	-	-	0.04±0.01	**0.03±0.02**	0.03±0.02
h^2_T	0.25	0.20	0.19	0.18	0.17	0.18
AIC	45463.888	45455.748	45457.194	45456.136	**45455.654 (best)**	45457.480
**TRAIT: BW40**						
σ^2_a	21214.4	**13091.3**	11679.4	15976.1	13077.5	11528.0
σ^2_m	-	**4277.19**	3820.60	-	4095.31	3359.41
σ_{am}	-	**-**	1176.01	-	-	1268.26
σ^2_c	-	**-**	-	2631.72	159.60	377.87
σ^2_e	32444.5	**35434.2**	36171.7	33966.7	35420.4	36200.0
σ^2_p	53758.9	**52802.7**	52847.6	52574.5	52752.8	52733.5
h^2	0.39±0.04	**0.25±0.05**	0.22±0.06	0.30±0.05	0.25±0.05	0.22±0.06
m^2	-	**0.08±0.02**	0.07±0.03	-	0.08±0.04	0.06±0.04
r_{am}	-	**-**	0.18±0.29	-	-	0.20±0.33
c^2	-	**-**	-	0.05±0.02	0.00±0.03	0.01±0.03
h^2_T	0.39	0.29	0.29	0.30	0.29	0.29
AIC	37580.224	**37567.692 (best)**	37569.308	37572.956	37569.68	37571.246
**TRAIT: EW28**						
σ^2_a	**7.01**	6.89	6.33	6.77	6.77	6.25
σ^2_m	**-**	0.08	0.02	-	0.001	0.01
σ_{am}	**-**	-	0.32	-	-	0.26
σ^2_c	**-**	-	-	0.18	0.18	0.15
σ^2_e	**9.58**	9.61	9.89	9.60	9.60	9.85
σ^2_p	**16.59**	16.58	16.56	16.55	16.55	16.53
h^2	**0.42±0.04**	0.42±0.05	0.38±0.06	0.41±0.05	0.41±0.05	0.38±0.07
m^2	**-**	0.01±0.02	0.005±0.02	-	0.005±0.02	0.001±0.02
r_{am}	**-**		0.97[€]	-	-	0.96[€]
c^2	**-**	-	-	0.014±0.024	0.014±0.030	0.020±0.031
h^2_T	0.42	0.42	0.41	0.41	0.41	0.40
AIC	**12751.626 (Best)**	12753.55	12755.144	12753.014	12755.016	12756.714

**TRAIT: EW40**						
σ^2_a	**6.92**	6.87	6.50	6.52	6.52	6.32
σ^2_m	**-**	0.03	0.008	-	0.001	0.003
σ_{am}	**-**	-	0.21	-	-	0.11
σ^2_c	**-**	-	-	0.22	0.22	0.20
σ^2_e	**13.61**	13.62	13.80	13.70	13.70	13.79
σ^2_p	**20.53**	20.52	20.52	20.44	20.44	20.44
h^2	**0.34±0.04**	0.34±0.05	0.32±0.06	0.32±0.05	0.32±0.05	0.31±0.06
m^2	**-**	0.001±0.02	0.000±0.02	-	0.000±0.02	0.000±0.02
r_{am}	**-**	-	0.93€	-	-	0.83€
c^2	**-**	-	-	0.01±0.01	0.01±0.02	0.01±0.02
h^2_T	0.34	0.34	0.33	0.32	0.32	0.32
AIC	**12920.63 (Best)**	12922.624	12924.466	12922.1	12924.104	12926.056
**TRAIT: EP40**						
σ^2_a	112.84	89.63	73.58	**64.01**	64.13	55.04
σ^2_m	-	13.84	9.35	**-**	0.004	0.75
σ_{am}	-	-	16.25	**-**	-	6.41
σ^2_c	-	-	-	**27.35**	27.33	25.64
σ^2_e	498.75	506.28	513.17	**511.19**	511.13	515.35
σ^2_p	611.58	609.75	612.36	**602.56**	602.59	603.19
h^2	0.18±0.03	0.15±0.04	0.12±0.04	**0.11±0.03**	0.11±0.03	0.09±0.04
m^2	-	0.02±0.01	0.02±0.02	**-**	0.000±0.01	0.001±0.0
r_{am}	-	-	0.62±0.69	**-**	-	0.99€
c^2	-	-	-	**0.05±0.02**	0.05±0.02	0.04±0.02
h^2_T	0.18	0.16	0.17	0.11	0.11	0.11
AIC	31414.33	31413.678	31414.58	**31405.828 (best)**	31407.828	31409.418
**TRAIT: EM40**						
σ^2_a	0.22	0.17	0.18	**0.15**	0.15	0.15
σ^2_m	-	0.04	0.04	**-**	0.01	0.01
σ_{am}	-	-	-0.008	**-**	-	-0.001
σ^2_c	-	-	-	**0.05**	0.04	0.04
σ^2_e	1.09	1.09	1.09	**1.09**	1.09	1.09
σ^2_p	1.31	1.31	1.31	**1.30**	1.30	1.30
h^2	0.17±0.03	0.13±0.04	0.14±0.05	**0.12±0.04**	0.11±0.04	0.11±0.05
m^2	-	0.03±0.02	0.03±0.02	**-**	0.01±0.029	0.01±0.02
r_{am}	-	-	-0.09±0.40	**-**	-	-0.01±0.91
c^2	-	-	-	**0.04±0.02**	0.03±0.02	0.03±0.02
h^2_T	0.17	0.15	0.14	0.12	0.12	0.12
AIC	4177.004	4175.952	4177.916	**4173.432 (best)**	4175.25	4177.25

Values in the parentheses are standard errors; Column in bold represents estimates from best model as per AIC
$^+\sigma^2_a$, σ^2_c, σ^2_m, σ^2_e and σ^2_p are additive direct, maternal permanent environmental, maternal genetic, residual variance and phenotypic variance, respectively; h^2, heritability; m^2, maternal heritability; r_{am}, direct-maternal genetic correlation; $c^2 = \sigma^2_c/\sigma^2_p$; h^2_t is total heritability; AIC, Akaike's Information Criterion for the model obtained from WOMBAT; € Indicates that the approximation used to define standard errors of parameter estimates failed.
